# Supplementary material for: Towards an uncertainty evaluation model for marine track considering local sampling cloud and sample information
Source: PLoS One. 2022 Jun 24;17(6):e0269184. doi: 10.1371/journal.pone.0269184 (PMC9231766; doi:10.1371/journal.pone.0269184)
Supplement: Attachment — Submitted filename: Response to Reviewers.doc [file pone.0269184.s002.doc]

**Journal: Plos One**

**Paper Title: Towards an uncertainty evaluation model for marine track considering local sampling cloud and sample information**

**Manuscript ID: PONE-D-21-23286**

**Date: 6 Dec., 2021**

Response to Reviewers

We are very grateful to Dr. Yiming Tang, the Academic Editor, the Reviewers, and the magazine staff for their constructive comments and suggestions to improve the quality of our manuscript.

The manuscript has been revised according to the comments, and the corresponding changes can be found in the file of ‘Revised Manuscript with Track Changes’. Detailed modifications of the manuscript and answers to the questions are listed as follows, where “**C**” refers to the word “**Comment**”, while “**R**” refers to “**Reply**”.

**#0 Response to Academic Editor’s comments**

**C0.1:** Please include the following items when submitting your revised manuscript:

- A rebuttal letter that responds to each point raised by the academic editor and reviewer(s). You should upload this letter as a separate file labeled 'Response to Reviewers'.
- A marked-up copy of your manuscript that highlights changes made to the original version. You should upload this as a separate file labeled 'Revised Manuscript with Track Changes'.
- An unmarked version of your revised paper without tracked changes. You should upload this as a separate file labeled 'Manuscript'.

**R0.1:** Thanks for your reminding. The three files will be uploaded when submitting the revised manuscript according to your requirements.

**C0.2:** If you would like to make changes to your financial disclosure, please include your updated statement in your cover letter. Guidelines for resubmitting your figure files are available below the reviewer comments at the end of this letter. If applicable, we recommend that you deposit your laboratory protocols in protocols.io to enhance the reproducibility of your results.

**R0.2:** Thanks for your advice. Since our simulation was carried out on the computer, there is no laboratory protocols. For other advice, we have done as required.

**#1 Response to Reviewer 1’s comments**

**C1.1:** The research paper was written well. Authors explained the cloud techniques in a good and understand way. Really reviewing this manuscript needs lot of patient. I appreciate the authors for used all formula.

**R1.1:** Thanks for your humorous comments.

**#2 Response to Reviewer 2’s comments**

**C2.1:** There is an area of improvement of the discussion section. The arguments in the discussion section is not supported by the any citation of the previous works.

**R2.1:** Thanks for your comment. We have improved the arguments in the discussion section according to your requirement.
**C2.2**: Less people are investigated, which may affect the precision of the evaluation result.

**R2.2:** Thanks for your comment. In order to test the effectiveness of the proposed comprehensive track evaluation model based on sample information, this study consulted some representative opinions of experts. On the other hand, an expert input optimization module was specially developed in our software system to open an access for dynamically improving the standard, thus increasing the accuracy and reasonability of the estimation results.

**#3 Response to Journal requirements**

**C3.1:** Please ensure that your manuscript meets PLOS ONE's style requirements, including those for file naming. The PLOS ONE style templates can be found at

https://journals.plos.org/plosone/s/file?id=wjVg/PLOSOne_formatting_sample_main_body.pdf and [https://journals.plos.org/plosone/s/file?id=ba62/PLOSOne_formatting_](https://journals.plos.org/plosone/s/file?id=ba62/PLOSOne_formatting_sample_title_authors_affiliations.pdf)

[sample_title_authors_affiliations.pdf](https://journals.plos.org/plosone/s/file?id=ba62/PLOSOne_formatting_sample_title_authors_affiliations.pdf).

**R3.1:** Thanks for your reminding. The manuscript has been checked carefully to satisfy the requirements in the two style templates.

**C3.2:** We note that you have provided funding information that is not currently declared in your Funding Statement. However, funding information should not appear in the Acknowledgments section or other areas of your manuscript. We will only publish funding information present in the Funding Statement section of the online submission form. Please remove any funding-related text from the manuscript and let us know how you would like to update your Funding Statement. Please include your amended statements within your cover letter; we will change the online submission form on your behalf.

**R3.2:** Thanks for your reminding. We have deleted funding-related text from the manuscript, and included the amended statements within the cover letter.

**C3.3:** In your Data Availability statement, you have not specified where the minimal data set underlying the results described in your manuscript can be found. Upon re-submitting your revised manuscript, please upload your study’s minimal underlying data set as either Supporting Information files or to a stable, public repository and include the relevant URLs, DOIs, or accession numbers within your revised cover letter. We will update your Data Availability statement to reflect the information you provide in your cover letter.

**R3.3:** Thanks for your reminding. Our study’s minimal data set will be uploaded upon re-submitting the revised manuscript as Supporting Information files. And the updated Data Availability statement has been provided in the cover letter.

**C3.4:** Please remove your figures from within your manuscript file, leaving only the individual TIFF/EPS image files, uploaded separately. These will be automatically included in the reviewers’ PDF. Please review your reference list to ensure that it is complete and correct. If you have cited papers that have been retracted, please include the rationale for doing so in the manuscript text, or remove these references and replace them with relevant current references. Any changes to the reference list should be mentioned in the rebuttal letter that accompanies your revised manuscript. If you need to cite a retracted article, indicate the article’s retracted status in the References list and also include a citation and full reference for the retraction notice.

**R3.4:** Thanks for your reminding. Figures have been removed from the manuscript file, and will be uploaded separately. The reference list has been checked carefully. Two master theses of ‘Gu X. (2014) Research on BRM assessment model based on the VDragon 3000 maritime simulator. Master, Dalian Maritime University (Item 15)’ and ‘Sheng Y. (2016) Research on automatic evaluation system of BPM based on marine simulator. Master, Jimei University (Item 20)’ have been deleted since no reference samples of degree theses can be found in Manuscript Body Formatting Guidelines, and the website of http://journals.plos.org/plosone/s/submissionguidelines#loc-references is not available for this moment. And the English version of Chinese article of ‘Guo L. (2015) Simulation and evaluation of NAVTEX receiver for navigation simulator (Item 17)’ is deleted since it is not found on the Internet. Other format and title changes to the references are marked in the file of ‘Revised Manuscript with Track Changes’.
